# Supplementary material for: OpenMM 7: Rapid development of high performance algorithms for molecular dynamics
Source: PLoS Comput Biol. 2017 Jul 26;13(7):e1005659. doi: 10.1371/journal.pcbi.1005659 (PMC5549999; doi:10.1371/journal.pcbi.1005659)
Supplement: S1 Source Code — (ZIP) [file pcbi.1005659.s002.zip › OpenMM-7.0.1-Source/docs/api-python/generated/simtk.openmm.app.amberinpcrdfile.AmberInpcrdFile.html]

AmberInpcrdFile — OpenMM 7.0.1.dev-5e86c4f documentation


# AmberInpcrdFile¶

*class* `simtk.openmm.app.amberinpcrdfile.``AmberInpcrdFile`(*file*, *loadVelocities=None*, *loadBoxVectors=None*)¶
:   AmberInpcrdFile parses an AMBER inpcrd file and loads the data stored in it.

    `__init__`(*file*, *loadVelocities=None*, *loadBoxVectors=None*)¶
    :   Load an inpcrd file.

        An inpcrd file contains atom positions and, optionally, velocities and
        periodic box dimensions.

        |  |  |
        | --- | --- |
        | Parameters: | - **file** (*str*) – The name of the file to load - **loadVelocities** (*bool*) – Deprecated. Velocities are loaded automatically if present - **loadBoxVectors** (*bool*) – Deprecated. Box vectors are loaded automatically if present |

    Methods

    |  |  |
    | --- | --- |
    | `__init__`(file[, loadVelocities, loadBoxVectors]) | Load an inpcrd file. |
    | `getBoxVectors`([asNumpy]) | Get the periodic box vectors. |
    | `getPositions`([asNumpy]) | Get the atomic positions. |
    | `getVelocities`([asNumpy]) | Get the atomic velocities. |

    `getPositions`(*asNumpy=False*)¶
    :   Get the atomic positions.

        |  |  |
        | --- | --- |
        | Parameters: | **asNumpy** (*bool=False*) – if true, the values are returned as a numpy array instead of a list of Vec3s |

    `getVelocities`(*asNumpy=False*)¶
    :   Get the atomic velocities.

        |  |  |
        | --- | --- |
        | Parameters: | **asNumpy** (*bool=False*) – if true, the vectors are returned as numpy arrays instead of Vec3s |

    `getBoxVectors`(*asNumpy=False*)¶
    :   Get the periodic box vectors.

        |  |  |
        | --- | --- |
        | Parameters: | **asNumpy** (*bool=False*) – if true, the values are returned as a numpy array instead of a list of Vec3s |

    `__delattr__`¶
    :   x.\_\_delattr\_\_(‘name’) <==> del x.name

    `__format__`()¶
    :   default object formatter

    `__getattribute__`¶
    :   x.\_\_getattribute\_\_(‘name’) <==> x.name

    `__hash__`¶

    `__reduce__`()¶
    :   helper for pickle

    `__reduce_ex__`()¶
    :   helper for pickle

    `__repr__`¶

    `__setattr__`¶
    :   x.\_\_setattr\_\_(‘name’, value) <==> x.name = value

    `__sizeof__`() → int¶
    :   size of object in memory, in bytes

    `__str__`¶

High performance molecular simulation on GPUs


### Navigation

- Application Layer
  - Loaders and Setup
  - Representation and Manipulation
  - Simulation
  - Reporting Output
  - Extras
- Library Layer

©2015, Stanford University and the Authors.
|
Powered by Sphinx 1.3.1
& Alabaster 0.7.7
|
Page source
